# Air Quality Monitoring in Schools: Evaluating the Effects of Ventilation Improvements on Cognitive Performance and Childhood Asthma

**DOI:** 10.7759/cureus.83306

**Published:** 2025-05-01

**Authors:** Victor C Ezeamii, Alex N Egbuchiem, Chekwube M Obianyo, Providence Nwoke, Lilian Okwuonu

**Affiliations:** 1 Public Health, Jiann-Ping Hsu College of Public Health, Georgia Southern University, Statesboro, USA; 2 Public Health Research, College of Public Health, University of Nebraska Medical Center, Omaha, USA; 3 Research and Development, Georgia Southern University, Statesboro, USA; 4 Public Health, University of Suffolk, Ipswich, GBR; 5 Nursing, Wayne County Community College District, Detroit, USA

**Keywords:** air quality monitoring, childhood asthma, cognitive performance, schools, ventilation improvements

## Abstract

In schools, indoor air quality (IAQ) is critical to students’ health, cognitive performance, and overall well-being. Poor ventilation can lead to increased exposure to airborne pollutants, exacerbating respiratory conditions, such as childhood asthma, while impairing concentration, memory, and academic performance. Poor IAQ contributes to nearly 14 million missed school days annually due to asthma-related complications and a 15% increase in asthma-related hospital visits among students. This review examines the impact of ventilation improvements on air quality, cognitive function, and asthma prevalence among school-aged children. It explores various air quality monitoring techniques, the effectiveness of ventilation upgrades, and the regulatory frameworks guiding indoor air quality in educational settings. Literature suggests that enhanced ventilation strategies, such as high-efficiency particulate air (HEPA) filtration and increased outdoor air exchange, significantly reduce indoor pollutants. These improvements correlate with better respiratory health outcomes and cognitive enhancements, demonstrating the need for evidence-based policies to promote optimal IAQ in schools. School administrators and policymakers can foster healthier learning environments that support student development and long-term well-being by integrating air quality monitoring with sustainable ventilation strategies. The review underscores the urgency of adopting cost-effective, scalable air quality interventions to mitigate health risks and enhance academic performance in educational institutions.

## Introduction and background

Importance of indoor air quality in schools

Indoor air quality (IAQ) in schools significantly affects students’ health, cognitive development, and academic performance. Poor IAQ contributes to several million missed school days annually due to asthma-related complications and a nearly 15% increase in asthma-related hospital visits among students. Poor IAQ, characterized by high levels of carbon dioxide (CO₂), particulate matter (PM), volatile organic compounds (VOCs), and allergens, has been linked to increased absenteeism, respiratory illnesses, and reduced cognitive function [1,2]. Inadequate ventilation exacerbates these issues, contributing to asthma prevalence and diminished learning outcomes [3].

Schools often experience elevated pollutant concentrations due to overcrowding, insufficient airflow, vehicular emissions, and exposure to industrial pollutants. The levels of indoor air pollutants are usually significantly higher than the levels outdoors, and students at various levels of cognitive development spend significant time in the classroom setting. Studies highlight the benefits of improved ventilation, smart air quality monitoring, and sustainability-driven designs in mitigating airborne contaminants while enhancing respiratory health and academic performance. Strategic IAQ interventions are particularly crucial in urban areas with high pollution exposure and rising childhood asthma rates [3]. Poor IAQ has been directly linked to an increase in adverse health outcomes in children of school age, further reinforcing the need for targeted interventions [4].

**Figure 1:**
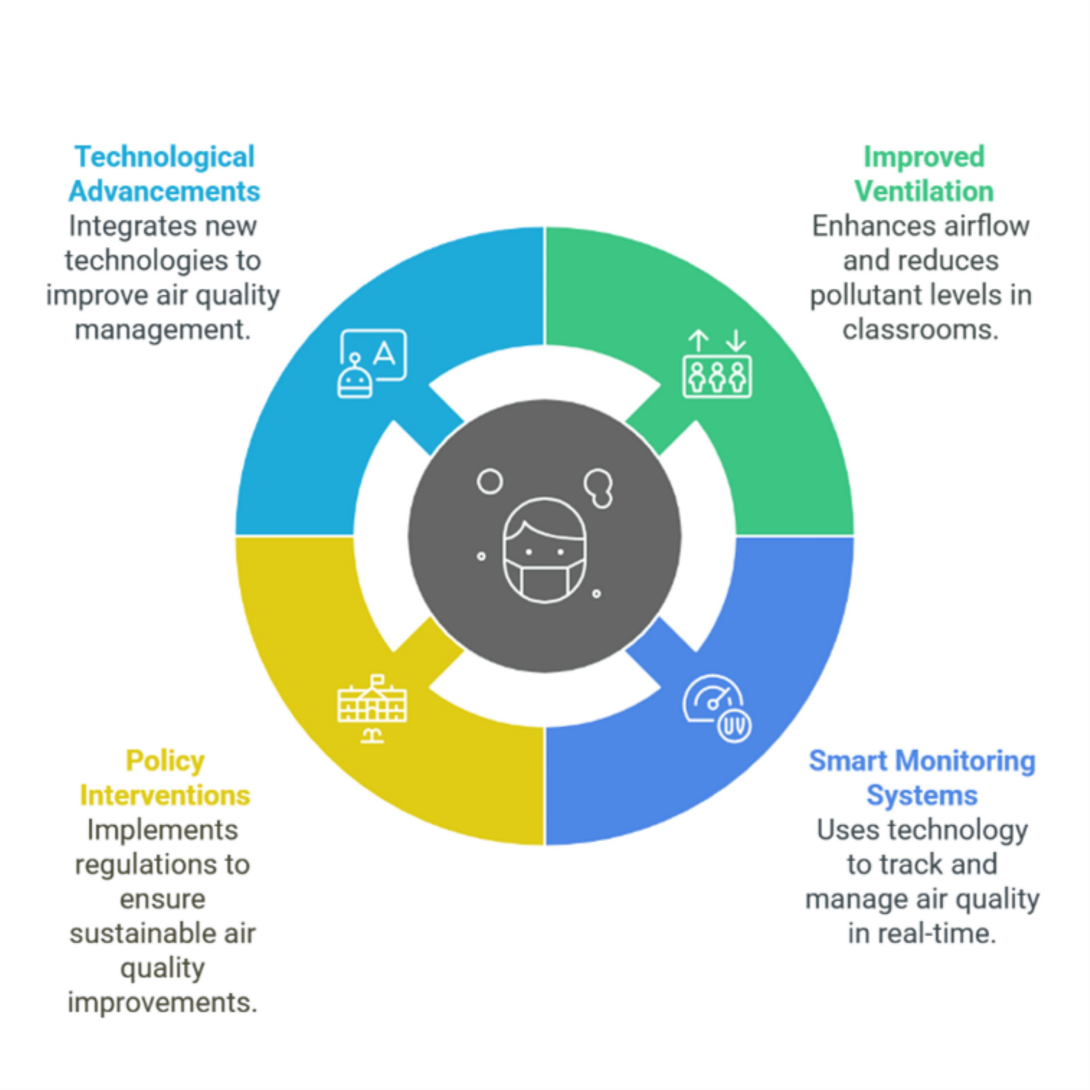
Interventions that improve general indoor air quality Image credit: Victor Ezeamii

IAQ interventions in schools encompass technological advancements, improved ventilation, smart monitoring systems, and policy regulations to enhance air quality management. Figure 1 illustrates key strategies for mitigating indoor pollutants and ensuring healthier student learning environments.

Poor IAQ also impairs cognitive function. High CO₂ levels, exceeding 1,000 parts per million (ppm), have been associated with a 10-20% decline in cognitive performance, while fine particulate matter (PM₂.₅) exposure increases asthma exacerbations by 15% [4-6]. In the U.S., approximately 14 million school days are lost annually due to asthma-related illnesses, underscoring the urgency of IAQ improvements [5].

Ventilation upgrades, such as compliance with American Society of Heating, Refrigerating, and Air-Conditioning Engineers (ASHRAE) Standard 62.1, which requires 5 L/s/person of outdoor air intake, have been shown to lower pollutant levels and enhance student well-being [7]. Classrooms with HEPA filters reduce airborne particulate concentrations by 60%, improving both respiratory health and cognitive function [1].

Policymakers and school administrators must prioritize cost-effective strategies such as increasing outdoor air exchange, utilizing low-emission building materials, and deploying real-time air quality sensors. Future research should explore long-term IAQ benefits, particularly in underprivileged schools where disparities are most pronounced. Addressing IAQ through ventilation improvements and environmental health policies is essential for fostering better student outcomes and long-term well-being.

Ventilation and its role in air quality

Ventilation is a fundamental component of IAQ management in schools, influencing pollutant concentrations and occupant health. It effectively reduces the indoor buildup of air pollutants. Proper ventilation reduces the accumulation of airborne contaminants, including CO₂, VOCs, PM, and microbial agents such as mold and bacteria [8]. Studies show that inadequate ventilation can lead to increased pollutant exposure, negatively impacting cognitive performance, student learning outcomes, and respiratory health [6]. Insufficient ventilation has been linked to higher absenteeism and the exacerbation of conditions such as asthma and allergic reactions [9]. Furthermore, poor IAQ can contribute to the spread of airborne infectious diseases, reinforcing the need for effective ventilation strategies in educational settings [10].

Types of Ventilation Systems in Schools

Ventilation in school buildings is typically categorized into natural, mechanical, and hybrid systems. Natural ventilation relies on outdoor air exchange through windows, vents, and passive airflows, which can be effective in regions with favorable climatic conditions. However, reliance on natural ventilation alone is often insufficient in high-density classrooms or urban environments with high outdoor pollution levels [11]. Jan et al. (2024) indicated that high levels of outdoor pollution, including PM₂.₅ and nitrogen dioxide (NO₂), can infiltrate naturally ventilated buildings, worsening indoor air conditions [12].

Mechanical ventilation, which includes heating, ventilation, and air conditioning (HVAC) systems, enables controlled air exchange and filtration, improving IAQ consistency. Schools with well-maintained HVAC systems that comply with the ASHRAE Standard 62.1, requiring a minimum of 5 liters per second per person (L/s/person) of outdoor air intake, report lower airborne contaminant levels and better student health outcomes [13]. Recent studies suggest that advanced HVAC systems incorporating high-efficiency particulate air (HEPA) filters and demand-controlled ventilation can significantly enhance IAQ while optimizing energy efficiency [14].

Hybrid ventilation, which integrates both natural and mechanical approaches, is increasingly being adopted to enhance energy efficiency while maintaining optimal air exchange rates. Research suggests that hybrid ventilation systems utilizing automated window operation and CO₂ sensors improve IAQ while reducing energy consumption by up to 30% compared to traditional mechanical ventilation alone [15]. Additionally, integrating real-time IAQ monitoring with hybrid ventilation strategies allows for adaptive control, ensuring that pollutant concentrations remain within safe thresholds [16].

**Figure 2:**
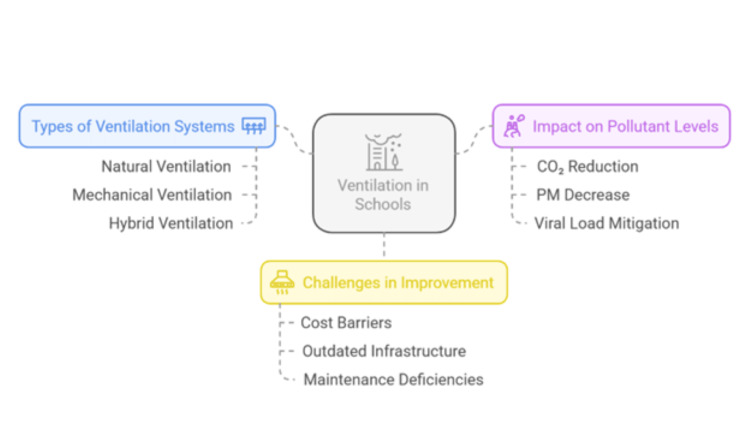
Ventilation in schools: systems, impacts, and challenges Image Credit: Victor Ezeamii

Ventilation in schools plays a crucial role in maintaining indoor air quality by reducing pollutants such as CO₂ and particulate matter. Figure 2 outlines different ventilation systems, their impact on air quality, and challenges such as cost barriers and infrastructure limitations.

Impact of Ventilation on Indoor Air Pollutant Levels

Ventilation improvements significantly influence pollutant concentrations in educational environments. For instance, a study by Yuejie Fan et al. (2023) found that increasing ventilation rates from 5 L/s/person to 10 L/s/person in classrooms led to a 50% reduction in CO₂ levels, improving cognitive performance among students [6]. Similarly, some other studies have demonstrated that schools implementing HEPA filtration in HVAC systems observed a 60% decrease in indoor PM₂.₅ levels, reducing asthma-related absenteeism by 25% [1,17].

Inadequate ventilation has also been linked to increased exposure to airborne pathogens. During the COVID-19 pandemic, poorly ventilated classrooms exhibited higher viral transmission rates due to the accumulation of aerosols in enclosed spaces. Studies have emphasized that increasing air exchange rates through advanced ventilation strategies reduced airborne viral loads by over 80%, reinforcing the critical role of ventilation in disease mitigation [18].

Challenges and Considerations in Ventilation Improvement

Despite the benefits of enhanced ventilation, many schools face challenges in upgrading their systems. Cost barriers, outdated infrastructure, and maintenance deficiencies contribute to suboptimal IAQ conditions. A national assessment revealed that over 41% of U.S. public schools have ventilation systems that fail to meet ASHRAE standards, exposing millions of students to poor air quality [19]. Addressing these deficiencies requires strategic investments in HVAC system upgrades, improved air filtration technologies, and real-time air quality monitoring.

Ventilation is crucial in maintaining indoor air quality (IAQ) in schools, directly affecting student health, cognitive performance, and overall well-being. Poor ventilation accumulates pollutants such as CO₂, PM, VOCs, and allergens, increasing respiratory illnesses and reducing academic outcomes.

Table 1 summarizes the key aspects of ventilation, including its impact on pollutant levels, challenges, interventions, and the role of technology in optimizing IAQ.

**Table 1:** Ventilation and indoor air quality in schools: challenges and solutions CO₂: carbon dioxide; VOCs: volatile organic compounds; PM: particulate matter; HVAC: heating, ventilation, and air conditioning; HEPA: high-efficiency particulate air; IAQ: indoor air quality; ASHRAE: American Society of Heating, Refrigerating, and Air-Conditioning Engineers

Aspect	Key Findings	Ventilation Strategies	Technology Solutions	Expected Outcomes
Role of Ventilation in IAQ	Ventilation reduces CO₂, VOCs, PM, and microbial agents, improving air quality and health [8].	Maintain proper air circulation to prevent pollutant buildup and improve student health.	Smart air monitoring sensors for real-time pollutant tracking.	Healthier indoor environments, reduced asthma cases, and improved student well-being.
Types of Ventilation	Natural, mechanical, and hybrid ventilation systems are used in schools, with HVAC systems providing the most control [11].	HVAC systems are used for controlled air exchange, and hybrid systems are used for energy efficiency.	Hybrid ventilation systems combine natural airflow and mechanical filtration.	More efficient air exchange, lower pollutant levels, and better temperature control.
Impact on Pollutant Levels	Increasing ventilation rates from 5 L/s to 10 L/s reduced CO₂ by 50%; HEPA filters decreased PM₂.₅ by 60% [1,6]	Adopt HEPA filtration and increase outdoor air intake to reduce contaminants.	HEPA in HVAC systems to reduce airborne pollutants.	Enhanced cognitive function, reduced absenteeism, and improved academic performance.
Health and Cognitive Effects	Poor IAQ increases respiratory illnesses (asthma, allergies) and reduces cognitive performance [20,21].	Monitor CO₂ and PM₂.₅ levels, ensuring adequate ventilation to boost cognitive function.	CO₂ and PM₂.₅ detection systems for proactive IAQ management.	Lower risk of respiratory diseases, fewer hospital visits, and improved learning outcomes.
Challenges & Considerations	41% of U.S. schools fail to meet ASHRAE ventilation standards due to outdated infrastructure and cost barriers [13].	Invest in HVAC upgrades, policy reforms, and maintenance programs to improve IAQ.	AI-driven predictive maintenance for ventilation system efficiency.	Long-term sustainability in school IAQ management, policy adherence, and reduced costs.

Optimizing school ventilation is a crucial strategy for improving IAQ, supporting cognitive development, and safeguarding student health. Schools must prioritize ventilation assessments, routine maintenance, and the adoption of cost-effective solutions, such as hybrid ventilation systems and HEPA filtration, to ensure a safe and conducive learning environment.

Health and cognitive effects of poor air quality

IAQ in schools significantly influences students' health, cognitive performance, and overall academic success. Exposure to pollutants such as CO₂, PM₂.₅, VOCs, and biological allergens has been linked to a higher prevalence of respiratory diseases, increased absenteeism, and cognitive impairments [1]. Poor IAQ is associated with an increased risk of asthma exacerbation and other respiratory conditions, particularly among children with pre-existing conditions [16,22]. Furthermore, long-term exposure to poor air quality in classrooms has been correlated with decreased student engagement and lower academic performance, as inadequate ventilation and high pollutant concentrations impair cognitive function [17,23].

Poor air quality in schools profoundly affects student health and academic performance, extending beyond immediate effects. Research suggests that prolonged exposure to elevated indoor CO₂ levels can reduce information retention and problem-solving abilities, contributing to long-term learning difficulties [13]. Additionally, high concentrations of airborne pollutants, such as PM₂.₅ and VOCs, have been associated with increased oxidative stress and neuroinflammation, potentially affecting brain development in young students [17]. Wenzhe Shang et al. also highlight the role of IAQ in behavioral outcomes, with evidence suggesting that poor air quality can increase fatigue, inattentiveness, and hyperactivity in students [17].

**Figure 3:**
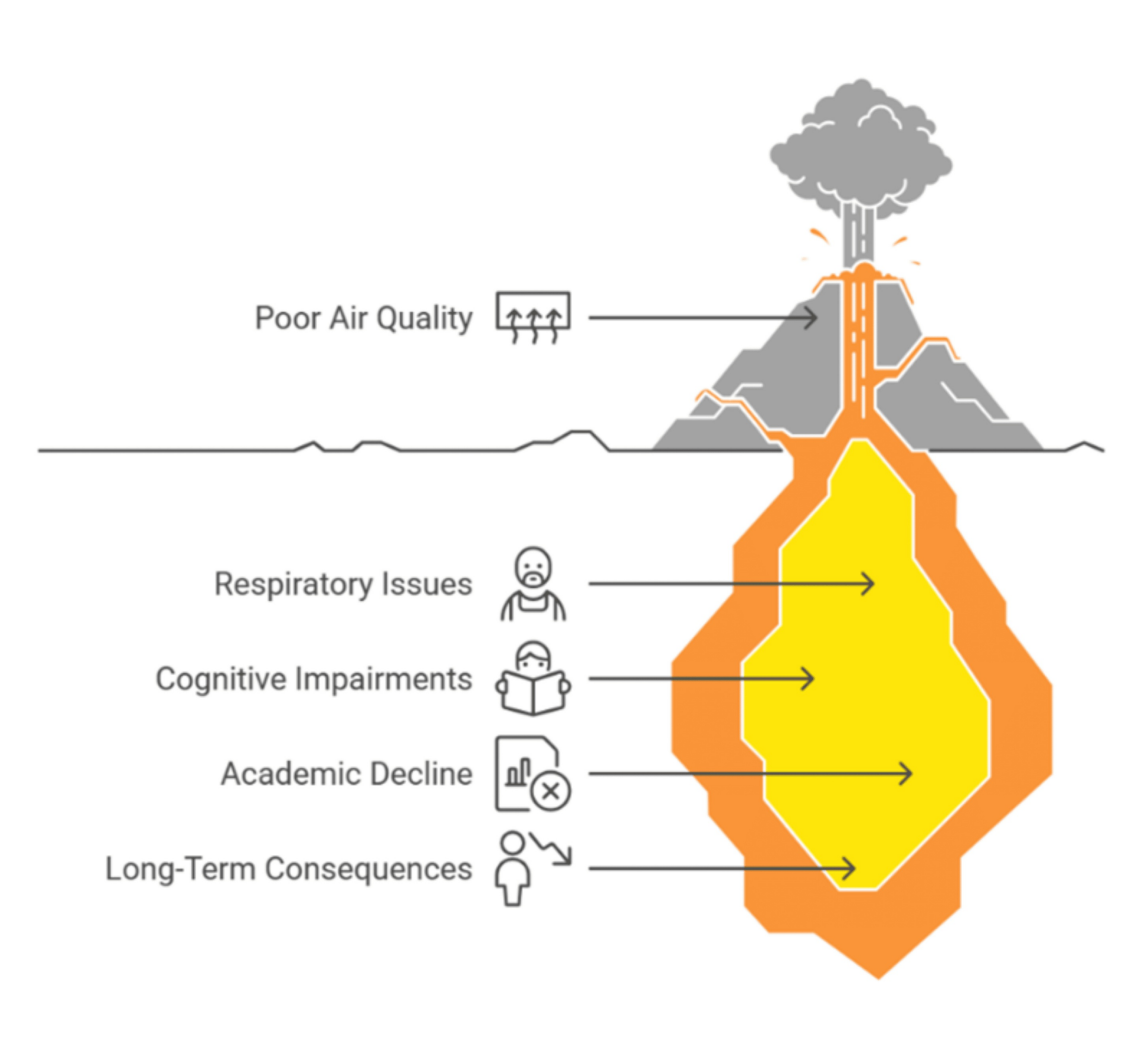
Unveiling the impact of poor air quality in schools Image credit: Victor Ezeamii

Figure 3 illustrates the hidden and long-term impacts of poor IAQ in schools, including respiratory issues, cognitive impairments, academic decline, and broader lifelong consequences. These findings emphasize the urgent need for improved ventilation strategies and pollutant control measures in school environments to safeguard student well-being and enhance educational outcomes.

Respiratory Health Effects of Poor Air Quality

Respiratory issues are among the most common health consequences of inadequate IAQ. Childhood asthma, a leading cause of school absenteeism in the United States, is exacerbated by airborne pollutants. The U.S. Environmental Protection Agency (EPA) estimates that poor IAQ contributes to nearly 14 million missed school days annually due to asthma-related complications [5]. Studies indicate that schools with higher PM₂.₅ levels report a 15% increase in asthma-related hospital visits among students [6]. Furthermore, high concentrations of NO₂ from indoor combustion sources and outdoor traffic emissions have been linked to a 30% higher risk of developing asthma in school-aged children [20].

Inadequate ventilation further exacerbates exposure to indoor allergens, such as mold, dust mites, and pet dander, which trigger allergic reactions and respiratory distress [8]. MacNaughton et al. (2015) concluded that classrooms with poor ventilation rates (CO₂ levels exceeding 1,500 ppm) report increased occurrences of coughing, wheezing, and other respiratory symptoms in students [1].

Cognitive Performance and Academic Outcomes

Beyond respiratory health, poor air quality has significant implications for cognitive function. Elevated CO₂ levels have been shown to impair students' attention, memory, and problem-solving skills. Fan et al. (2023) conducted a study in U.S. elementary schools and found that CO₂ concentrations above 1,000 ppm resulted in a 20% decline in cognitive test scores among students [6]. Similarly, a Harvard study reported that reducing CO₂ levels to 600 ppm improved decision-making abilities by 50% [1]. Other research has corroborated these findings, demonstrating that elevated CO₂ levels impair neurophysiological processes by reducing cerebral oxygenation, thereby affecting cognitive flexibility and reaction time [24-26]. 

Exposure to PM₂.₅ has also been linked to reduced academic performance. Mendel et al. (2021) analyzed standardized test scores from 1,200 schools and found that a 10 µg/m³ increase in PM₂.₅ levels correlated with a 6% decrease in math and reading scores. Moreover, long-term exposure to PM₂.₅ has been associated with neuroinflammation, oxidative stress, and reduced synaptic plasticity, which negatively impact cognitive function and learning ability [2]. Additionally, schools in high-traffic urban areas with increased air pollution have shown higher rates of cognitive fatigue and reduced student productivity compared to schools with lower pollution levels [22].

Neurotoxic pollutants, including lead and benzene, contribute to cognitive impairments. A study found that chronic exposure to indoor pollutants affects brain development in children, reducing working memory and executive function skills necessary for academic success. Furthermore, exposure to nitrogen dioxide (NO₂) and polycyclic aromatic hydrocarbons (PAHs) has been linked to increased risks of attention deficit hyperactivity disorder (ADHD) and other neurodevelopmental disorders in school-age children [24,27]. New evidence suggests that even low-level exposure to indoor toxins can alter neural connectivity and impact long-term cognitive resilience, making effective air quality management essential in school environments [24]. 

Long-Term Implications and Policy Recommendations

Long-term exposure to poor IAQ during childhood has been linked to chronic respiratory diseases, developmental disorders, and reduced educational attainment. Mendell et al. (2013) highlighted that students exposed to suboptimal IAQ throughout their academic years have a 30% higher likelihood of developing chronic lung diseases in adulthood [22]. Given these significant health and cognitive effects, there is an urgent need for evidence-based policies to improve ventilation and air quality monitoring in schools. Table 2 summarizes recommendations to reduce adverse outcomes related to indoor air contaminants.

**Table 2:** Health and cognitive impacts of poor air quality in schools CO₂: carbon dioxide; VOCs: volatile organic compounds; PM: particulate matter; HEPA: high-efficiency particulate air; IAQ: indoor air quality

Aspect	Key Findings	Effects on Students	Recommended Solutions	Expected Outcomes
Impact on Health & Academics	Exposure to CO₂, PM₂.₅, VOCs, and allergens increases respiratory diseases and cognitive impairments [1].	Increased absenteeism, reduced concentration, and poor academic performance.	Improve air circulation, regulate pollutant levels, and enhance IAQ monitoring.	Healthier school environments, reduced respiratory diseases, and better student performance.
Respiratory Health Effects	Poor IAQ contributes to 14M missed school days annually due to asthma; PM₂.₅ exposure increases hospital visits by 15% [6,21].	Higher risk of asthma, respiratory distress, and hospitalizations.	Use air purifiers, increase ventilation rates, and control indoor allergens.	Lower asthma rates, improved air quality, and fewer respiratory illnesses.
Cognitive Performance	CO₂ levels above 1,000 ppm cause a 20% decline in cognitive scores; reducing CO₂ to 600 ppm improves decision-making by 50% [1].	Impaired memory, problem-solving skills, and attention span.	Monitor CO₂ levels, reduce exposure to NO₂ and VOCs, and integrate innovative air systems.	Enhanced focus, better cognitive function, and increased academic success.
Long-Term Implications	Students exposed to poor IAQ have a 30% higher risk of chronic lung disease and developmental disorders [22].	Long-term developmental issues, and a higher risk of chronic illnesses.	Implement health screenings, regulate indoor air policies, and educate school administrators.	Long-term student well-being, fewer chronic diseases, and better public health.
Policy & Interventions	HEPA filters, real-time air monitoring, and improved ventilation reduce absenteeism by 40% and enhance academic scores by 10% [20].	Better academic performance, reduced health risks, and improved learning conditions.	Adopt energy-efficient ventilation, enforce IAQ policies, and fund school infrastructure upgrades.	Sustainable school air quality management and long-term educational benefits.

Strategies such as increasing air exchange rates, utilizing HEPA filters, and implementing real-time air quality monitoring systems have demonstrated substantial benefits. Schools that adopted these measures reported a 40% reduction in absenteeism due to respiratory illnesses and a 10% improvement in cognitive test scores [20]. Integrating these interventions into school infrastructure can foster healthier learning environments, ensuring students achieve their full academic potential while minimizing health risks. This study aims to evaluate the effect of ventilation improvements on IAQ in schools, focusing on a reduction of pollutants and the potential impact on students' health and academic performance.

## Review

Methodology

This study employed a comprehensive narrative review methodology to explore the relationship between IAQ, ventilation improvements, cognitive performance, and childhood asthma in school environments. Relevant literature was sourced using academic databases, including PubMed, Google Scholar, and Research Gate, focusing on peer-reviewed articles published between 2010 and 2024.

The inclusion criteria for this review were as follows: (1) studies examining the role of ventilation in influencing IAQ in school settings, (2) research evaluating cognitive performance or asthma outcomes relating to indoor pollutant exposure, and (3) articles assessing monitoring technologies for air quality in educational environments. Only articles written in English and providing full-text access were considered. Studies focused solely on residential or occupational settings were excluded unless they provided comparative insights applicable to schools.

**Figure 4:**
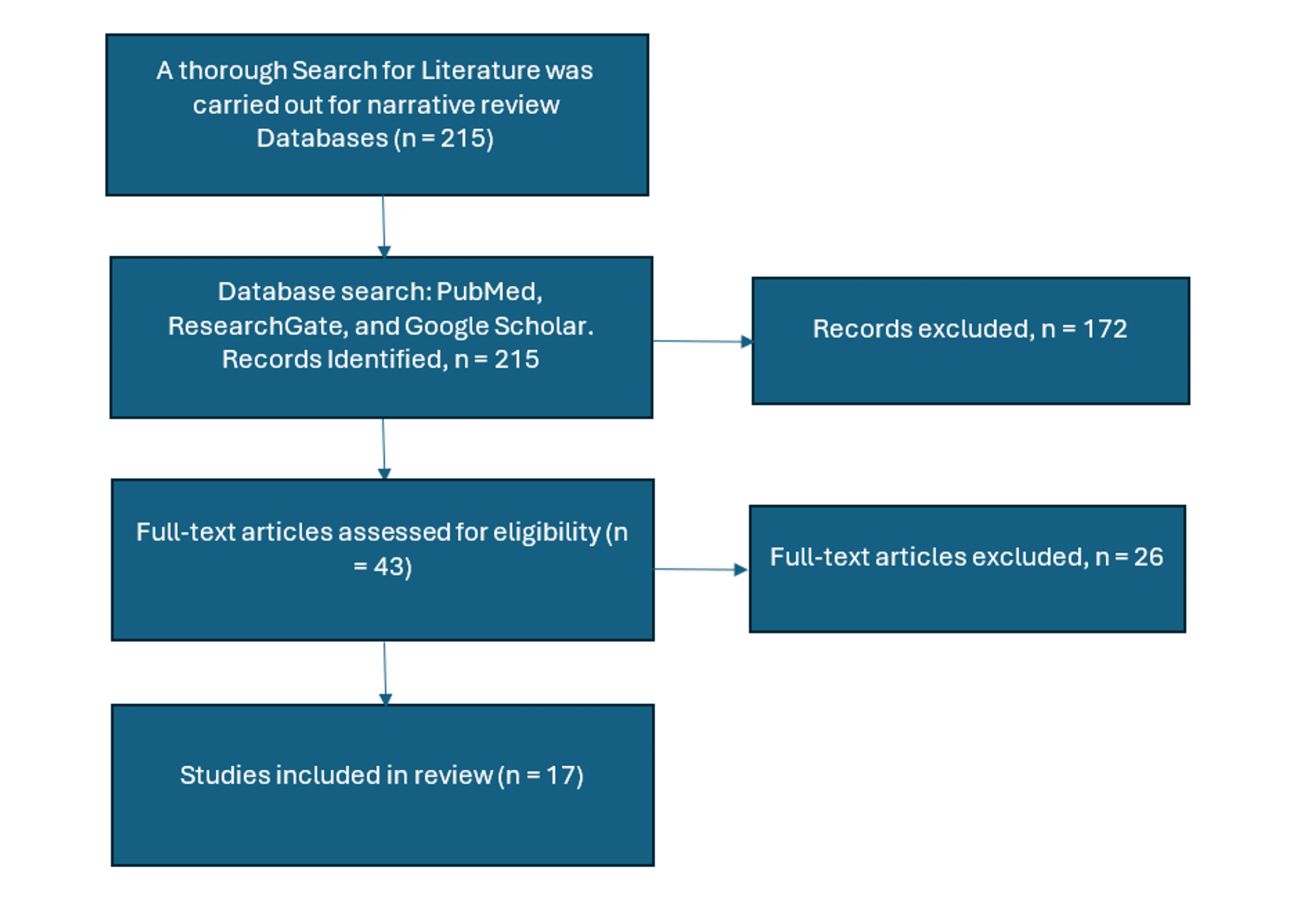
Methodology of the narrative review Image Credit: Victor Ezeamii

A structured three-phase approach was used: first, duplicate records were eliminated; second, titles and abstracts were screened for relevance; and third, eligible full-text articles were reviewed for methodological rigor and relevance to IAQ indicators such as CO₂, PM₂.₅, VOCs, and associated health and cognitive outcomes. Data on intervention types (e.g., mechanical, hybrid ventilation), pollutant measurement strategies (e.g., traditional sampling, sensor-based, Internet of Things (IoT)), and quantifiable outcomes (e.g., absenteeism rates, cognitive test scores, asthma prevalence) were extracted. See Figure 4.

Methods of air quality monitoring in schools

Effective school air quality monitoring is essential for ensuring a healthy learning environment, as poor indoor air quality (IAQ) can adversely affect students' health and academic performance. Various methods, from traditional approaches to modern sensor-based technologies, have been developed to assess IAQ in educational settings.

Traditional Monitoring Techniques

Historically, schools' indoor air quality (IAQ) assessments have relied on periodic sampling and laboratory analysis. This method involves collecting air samples over a specific period and analyzing them for pollutants such as PM, VOCs, CO₂, and other contaminants. While this approach provides accurate and detailed information, it is often resource-intensive, requiring specialized equipment and trained personnel. Additionally, the time lag between sample collection and analysis can delay identifying air quality issues, limiting the ability to implement timely interventions [28]. 

Traditional air sampling methods, such as gravimetric analysis for PM and gas chromatography-mass spectrometry (GC-MS) for VOC detection, have been widely used to characterize IAQ in school environments. However, studies indicate that these conventional techniques often fail to capture real-time variations in pollutant concentrations, making it challenging to assess short-term exposure risks effectively [29]. Furthermore, a limited sampling frequency can lead to underestimating peak pollution events, particularly in classrooms with fluctuating occupancy levels and seasonal changes in ventilation patterns [30]. 

Advancements in IAQ monitoring now emphasize real-time air quality sensing, leveraging low-cost sensors and wireless monitoring networks to provide continuous data on pollutant levels. Research shows that deploying networked sensor systems in schools significantly enhances IAQ assessment accuracy and allows for proactive mitigation strategies [31]. Additionally, studies have demonstrated that integrating artificial intelligence (AI) and machine learning algorithms with IAQ sensors improves the predictive modeling of air quality trends, enabling school administrators to anticipate and respond to pollution spikes more efficiently [32]. These modern approaches reduce operational costs and facilitate rapid decision-making, ensuring healthier indoor environments for students and staff.

Advancements in Sensor-Based Monitoring

Recent advancements in sensor technology have led to the development of low-cost, real-time monitoring devices that offer continuous assessment of IAQ parameters. These sensors can measure various pollutants, including PM₂.₅, CO₂, temperature, and humidity, providing immediate feedback on indoor environmental conditions. For instance, a study conducted in Portuguese classrooms utilized low-cost sensors to monitor IAQ during teaching hours, revealing significant exceedances in temperature and PM₁₀ levels [28]. The deployment of such sensors enables schools to promptly identify and address air quality issues, enhancing the overall learning environment.

Integration of IoT and Data Analytics

Integrating the IoT technology with air quality sensors has enhanced monitoring capabilities. IoT-enabled devices can transmit real-time data to centralized platforms, facilitating continuous surveillance and analysis. In a large-scale study across German schools, novel sensor instruments were installed in 329 classrooms to monitor CO₂ levels, sound, temperature, and humidity. The data collected were transmitted via Wi-Fi for further statistical evaluation, providing a comprehensive overview of the air quality situation over six months [33]. This approach allows for identifying patterns and trends, promptly supporting informed decision-making for IAQ management.

Community-Based Monitoring Initiatives

Community-based monitoring initiatives have also gained traction, emphasizing the role of local stakeholders in IAQ assessment. For example, a proposal to install air quality monitors in all 64,311 elementary schools in the United States aims to provide widespread and reliable air quality information. Affordable monitors like those from PurpleAir (Draper, Utah, US) can offer real-time data, enhancing community awareness and education on air pollution [34]. Such initiatives empower schools and communities to maintain healthy indoor environments proactively.

Challenges and Considerations

Despite the advancements in monitoring technologies, several challenges persist. Ensuring the accuracy and reliability of low-cost sensors is crucial, as discrepancies can lead to misinformed decisions. Proper calibration against reference equipment is essential to maintaining data integrity [28]. Additionally, the variability in classroom sizes, ventilation systems, and occupancy levels necessitates tailored monitoring approaches to effectively assess IAQ in diverse educational settings [33].

**Figure 5:**
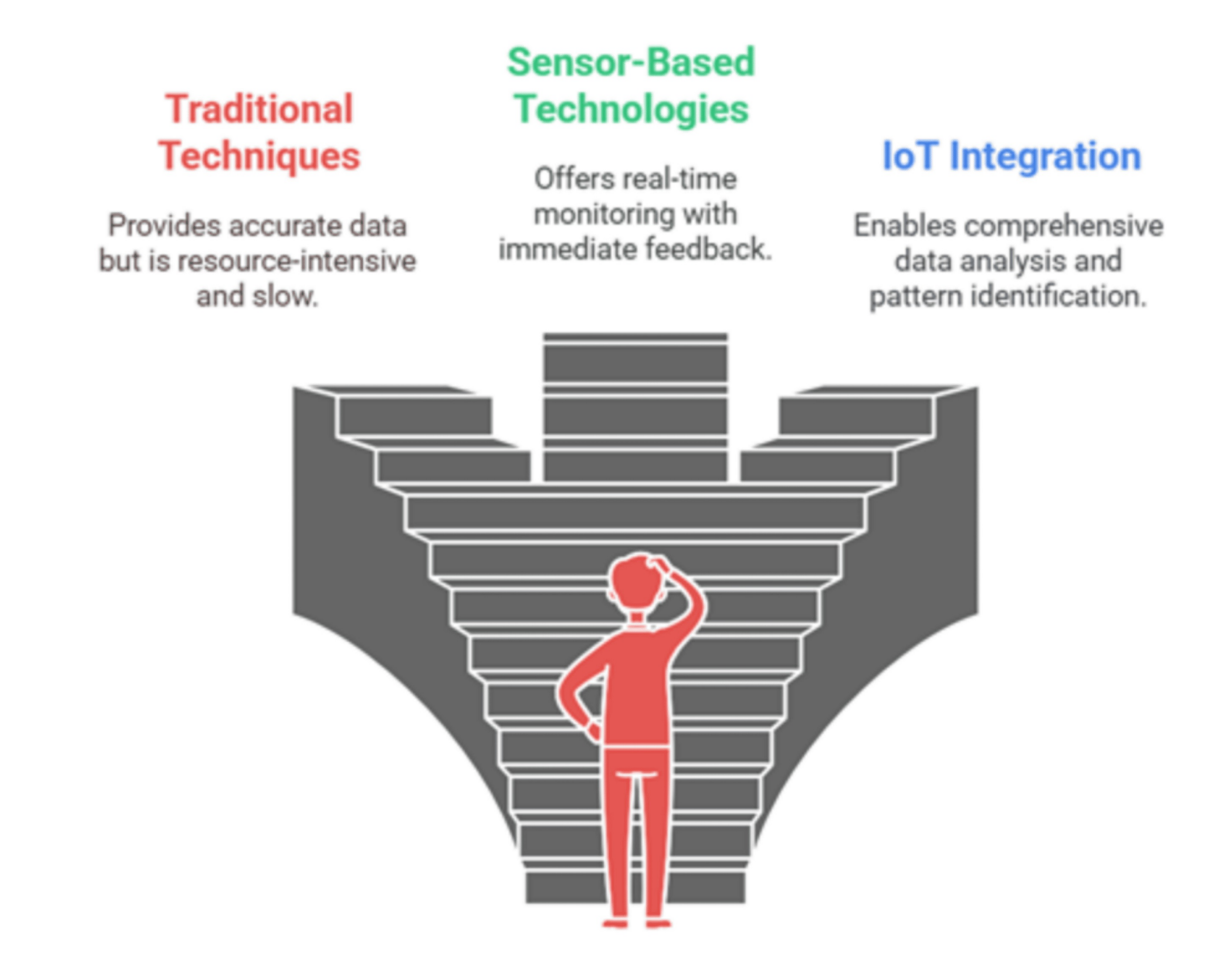
Choosing the best air quality monitoring method for schools: a comparative analysis Image credit: Victor Ezeamii

Selecting the appropriate air quality monitoring method in schools is crucial for effective IAQ management. Figure 5 compares traditional techniques, sensor-based technologies, and IoT integration, highlighting their advantages, limitations, and role in real-time air quality assessment.

Monitoring IAQ in schools is essential for maintaining a healthy learning environment and reducing exposure to pollutants that affect student health and academic performance. Traditional sampling methods provide precise but delayed results, while modern sensor-based and IoT-enabled technologies offer real-time monitoring solutions. Table 3 summarizes different IAQ monitoring methods and their advantages, limitations, and expected outcomes in school settings.

**Table 3:** Methods for monitoring air quality in schools: approaches, challenges, and outcomes CO₂: carbon dioxide; VOCs: volatile organic compounds; PM: particulate matter; IAQ: indoor air quality; IoT: Internet of Things

Monitoring Method	Description	Advantages	Limitations	Expected Outcomes
Traditional Sampling & Laboratory Analysis	Collects air samples to analyze pollutants like PM, VOCs, and CO₂. Accurate but time-consuming and resource-intensive [28].	Provides detailed pollutant analysis with high accuracy.	Time delays in results require specialized personnel and high costs.	Accurate identification of pollutants for effective IAQ interventions.
Sensor-Based Monitoring	Uses low-cost real-time sensors to measure pollutants such as PM₂.₅, CO₂, temperature, and humidity [28]	Enables continuous IAQ assessment with immediate feedback.	Sensor reliability may vary; requires periodic calibration.	Improved air quality management through real-time detection.
IoT and Data Analytics Integration	IoT-enabled sensors transmit real-time air quality data for statistical analysis and long-term monitoring [33].	Allows remote tracking, pattern recognition, and data-driven decision-making.	Depends on internet connectivity and data security measures.	Efficient IAQ policies and preventive measures in schools.
Community-Based Monitoring	Community-led initiatives use affordable monitors like PurpleAir to track school air pollution [34].	Increase community involvement and awareness of IAQ issues.	Limited data accuracy compared to professional monitoring tools.	Empowered schools and communities with real-time IAQ insights.
Challenges & Considerations	The accuracy of low-cost sensors, the need for calibration, and the variability in school environments pose challenges [28].	Enhances monitoring methods but requires proper calibration and tailored approaches.	Discrepancies in sensor reading may lead to misinformed actions.	Better implementation of tailored monitoring strategies for diverse school environments.

The evolution of air quality monitoring methods in schools, from traditional sampling to advanced sensor-based and community-driven approaches, has significantly enhanced the ability to maintain healthy indoor environments. Continuous monitoring and real-time data analysis enable timely interventions, safeguarding students' health and optimizing academic performance.

Impact of ventilation improvements on indoor air quality

Enhancing school ventilation systems is pivotal for improving IAQ, directly influencing students' health and academic performance. Effective ventilation strategies reduce the concentration of indoor pollutants, including carbon dioxide (CO₂), particulate matter (PM), and volatile organic compounds (VOCs), thereby creating a healthier learning environment.

Case Studies of Ventilation Upgrades

A comprehensive case study conducted in a newly constructed school building with reported IAQ issues examined the effects of upgrading to a supply air fan-assisted hybrid ventilation system. The study found that this improvement led to significant enhancements in both measured and perceived IAQ, highlighting the effectiveness of such systems in educational settings [35].

In another instance, Denver Public Schools undertook a project to update and adjust their ventilation systems to align with new safety guidelines to maximize protection against airborne diseases. This initiative involved comprehensive inspections and modifications of existing HVAC systems, improving air circulation and reducing the potential for airborne contaminant accumulation [36].

Quantitative Benefits of Improved Ventilation

Research indicates that increasing ventilation rates can lead to measurable student health and performance benefits. For example, doubling the ventilation rate from approximately 7.5 to 15 cubic feet per minute per person (cfm/person) has been associated with an 8% improvement in academic performance. Additionally, every 2 cfm/person increase in ventilation rate from 2 to 15 cfm/person corresponded to a 3% increase in the proportion of fifth-grade students passing standardized math and reading tests [5].

Challenges and Considerations

While the benefits of improved ventilation are evident, schools often face challenges in implementing these upgrades. Financial constraints, aging infrastructure, and the need for regular maintenance can impede efforts to enhance ventilation systems. Moreover, increasing ventilation rates may lead to higher energy consumption, necessitating a balance between IAQ improvements and energy efficiency [37].

Improving school ventilation enhances IAQ, reduces pollutant concentrations, and supports student health and academic performance. Case studies and research highlight the benefits of upgraded HVAC systems, increased air exchange rates, and hybrid ventilation solutions. Table 4 summarizes the role of ventilation improvements, their impact on students, associated challenges, and recommended strategies for maintaining a healthy learning environment.

**Table 4:** The impact of ventilation improvements on indoor air quality in schools CO₂: carbon dioxide; VOCs: volatile organic compounds; PM: particulate matter; IAQ: indoor air quality; ASHRAE: American Society of Heating, Refrigerating, and Air-Conditioning Engineers

Aspect	Key Findings	Effects on Students	Recommended Solutions	Expected Outcomes
Role of Ventilation in IAQ	Effective ventilation reduces CO₂, PM, and VOCs, improving IAQ and student health [35].	Healthier learning environments, reduced respiratory issues, and enhanced cognitive performance.	Adopt mechanical and hybrid ventilation systems to ensure proper air circulation.	Improved IAQ, healthier students, and reduced asthma-related absenteeism.
Case Studies of Ventilation Upgrades	Hybrid ventilation upgrades improved IAQ and air circulation in schools [35]. Denver Public Schools enhanced HVAC systems to reduce airborne contaminants [36].	Better air circulation, fewer airborne contaminants, and reduced transmission of diseases.	Regular maintenance of HVAC systems and compliance with safety guidelines.	Sustained air quality improvements and compliance with air safety guidelines.
Quantitative Benefits	Doubling ventilation rates from 7.5 to 15 cfm/person led to an 8% improvement in academic performance; a 2 cfm/person increase boosted standardized test pass rates by 3% [21].	Higher standardized test scores, improved concentration, and reduced absenteeism.	Increase ventilation rates in line with ASHRAE standards to enhance learning outcomes.	Stronger academic performance and higher student engagement.
Challenges & Considerations	Financial constraints, outdated infrastructure, and energy consumption concerns challenge ventilation improvements [37].	Risk of increased costs, higher energy consumption, and the need for policy support.	Implement energy-efficient ventilation technologies to balance IAQ improvements with cost savings.	Energy-efficient ventilation systems with optimized cost-effectiveness.
Strategic Recommendations	Investing in ventilation upgrades enhances IAQ, cognitive function, and academic outcomes, requiring strategic resource allocation.	Long-term academic and health benefits, improved school air quality policies.	Secure funding for ventilation enhancements and integration of IAQ monitoring technologies.	Comprehensive school IAQ policies and sustainable air quality improvements.

Investing in ventilation improvements is essential for promoting better IAQ in schools. Such enhancements mitigate health risks associated with poor air quality and contribute to improved cognitive function and academic success among students. Strategic planning and resource allocation are crucial to overcoming challenges and implementing effective ventilation solutions in educational institutions.

Effects of air quality on cognitive performance

IAQ significantly influences cognitive functions, particularly in learning environments like schools. Exposure to elevated levels of indoor pollutants, including CO₂, PM, and VOCs, has been linked to declines in cognitive performance among students.

Impact of CO₂ on Cognitive Function

Elevated CO₂ concentrations in classrooms are associated with decreased cognitive abilities. A study found that moderate increases in CO₂ levels, even within commonly observed indoor ranges, can impair decision-making performance [25]. Specifically, CO₂ concentrations of 1,000 ppm led to significant reductions in 6 out of 9 scales of decision-making performance, with further declines at 2,500 ppm. These findings suggest that typical indoor CO₂ levels can adversely affect cognitive functions critical for learning.

Effects of PM on Cognitive Performance

Exposure to PM₂.₅ has been linked to cognitive impairments. Research demonstrated that increased PM₂.₅ levels in classrooms correlate with reduced student attention and memory [38]. The study observed that a 10 µg/m³ increase in PM₂.₅ concentration was associated with a significant decrease in students' test scores, highlighting the detrimental impact of particulate pollution on cognitive performance [38].

Influence of VOCs on Cognitive Abilities

VOCs emitted from building materials, furnishings, and cleaning products can impair cognitive functions. Allen et al. conducted a study examining the effects of VOCs on cognitive performance and found that participants in environments with reduced VOC levels performed 61% better on cognitive tasks than those in conventional office environments [39]. This underscores the importance of managing VOC concentration in indoor environments to support cognitive health.

Comprehensive Impact of IAQ on Academic Performance

The cumulative effect of poor IAQ on various pollutants can lead to significant declines in academic performance. A review by Mendell and Heath concluded that inadequate ventilation and elevated indoor pollutant levels are consistently associated with diminished student performance. The review emphasized that improving IAQ through enhanced ventilation and pollutant source control can lead to measurable improvements in students' cognitive functions and academic achievements [2].

Mechanisms underlying IAQ-related cognitive impairments

The adverse effects of poor IAQ on cognitive performance are thought to be mediated through several physiological mechanisms. Elevated CO₂ levels can lead to hypercapnia, reducing cerebral oxygenation and impairing neuronal function [25]. Studies have shown that CO₂ concentrations exceeding 1,000 ppm can negatively impact cognitive flexibility, decision-making, and information retention, potentially hindering academic performance [6].

Exposure to particulate matter (PM₂.₅) and volatile organic compounds (VOCs) has also been linked to neuroinflammation and oxidative stress, both of which can disrupt neural pathways involved in cognition [38, 39] Neurotoxic pollutants such as nitrogen dioxide (NO₂) and polycyclic aromatic hydrocarbons (PAHs) have been associated with impaired executive function and increased risk of attention-related disorders in children [27]. Moreover, long-term exposure to air pollution can accelerate cognitive decline by affecting synaptic plasticity and neurotransmitter balance [26]. 

Maintaining optimal IAQ in educational settings is crucial for preserving and enhancing cognitive performance among students. A study indicated that improving ventilation and reducing airborne pollutants can significantly improve academic performance, particularly in standardized testing environments [23]. Additionally, real-time IAQ monitoring and adaptive ventilation strategies have been found to mitigate cognitive impairments associated with poor air quality, ensuring a healthier and more productive learning environment [24]. Implementing these strategies can significantly benefit students' learning outcomes and overall well-being. 

Childhood asthma and school air quality

IAQ in schools plays a critical role in the health and well-being of students, particularly those with asthma. Asthma is a chronic respiratory condition characterized by airway inflammation and hyperreactivity, leading to wheezing, coughing, and shortness of breath. Exposure to indoor pollutants can exacerbate these symptoms, resulting in increased absenteeism and decreased academic performance.

Prevalence of Asthma in School-Aged Children

Asthma is one of the most common chronic diseases among children. According to the Centers for Disease Control and Prevention (CDC), approximately 4.5 million children in the United States have asthma, accounting for about 6.5% of the pediatric population [40]. This prevalence underscores the importance of maintaining optimal IAQ in schools to manage and mitigate asthma symptoms among students.

Indoor Pollutants and Asthma Triggers

Several indoor pollutants commonly found in school environments, including PM, VOCs, mold and moisture, and allergens, can trigger asthma. PM contains fine particles from dust, dirt, and combustion processes that can irritate the respiratory system. VOCs are mainly emitted from building materials, cleaning agents, and furnishings in indoor spaces and can exacerbate asthma symptoms. Damp environments promote mold growth, releasing spores that can trigger asthma attacks. Allergens generated from dust mites, pet dander, and pest droppings are common school allergens that can worsen asthma.

Impact of Poor IAQ on Asthma Outcomes

Exposure to suboptimal IAQ in schools has been linked to several adverse outcomes for children with asthma, including increased absenteeism. Asthma-related absences are a significant concern. The EPA reports that asthma accounts for approximately 14 million missed school days annually. Frequent absences and health-related distractions can impair learning and academic achievement. Poor IAQ can lead to more frequent asthma exacerbations, increasing healthcare provider visits, and accumulating higher medical costs.

Strategies to Improve IAQ and Manage Asthma in Schools

Effective IAQ management programs are essential to reduce asthma triggers in school settings. The EPA's IAQ Tools for Schools program recommends several strategies. Regular maintenance of HVAC systems ensures proper ventilation and filtration to reduce pollutant levels. Moisture control is an important strategy for improving IAQ by addressing leaks and promptly repairing water-damaged areas so that mold growth is prevented. Minimizing the use of pesticides and managing pest populations through preventive measures is important. The use of low-emission materials, including selecting building materials and furnishings that emit low levels of VOCs, is another important factor. A study in the International Journal of Environmental Research and Public Health demonstrated that schools implementing comprehensive IAQ management programs observed a significant reduction in asthma-related incidents among students [41]. Maintaining good IAQ in schools is vital for managing asthma among students. By identifying and mitigating indoor pollutants, schools can create healthier environments that support all students' well-being and academic success, particularly those with asthma.

Global policy recommendations for enhancing indoor air quality in schools

Improving IAQ in schools is essential for safeguarding student health and optimizing academic performance. Effective policies should encompass comprehensive strategies that address ventilation, filtration, maintenance, and monitoring. Schools should develop and implement IAQ management plans that include regular inspection, maintenance, and operation of ventilation systems. The EPA emphasizes the importance of a coordinated approach to maintaining good IAQ, which involves educating building occupants and establishing routine inspection and maintenance policies. Investing in modern HVAC systems with HEPA filters can significantly reduce indoor pollutants. A study by Gaihre et al. (2014) published in the Journal of School Health found that improved ventilation and air filtration in schools are associated with better health outcomes and enhanced student academic performance [4]. Continuous monitoring of IAQ parameters, such as CO₂ levels, PM, and humidity, enables the timely identification and mitigation of air quality issues. Research by MacNaughton et al. (2015) in the International Journal of Environmental Research and Public Health highlights the effectiveness of real-time monitoring in maintaining optimal IAQ in educational settings [1]. Establishing clear IAQ policies and standards at the school or district level ensures consistent practices in maintaining air quality. The Environmental Law Institute underscores the need for state and local policies that set minimum IAQ standards and provide guidelines for schools to follow [42]. Allocating financial resources for IAQ initiatives is crucial. Federal funding remains available to school districts to support ventilation improvements. Public health departments can encourage K-12 school officials to use available funding to improve ventilation and help reduce the transmission of respiratory diseases in K-12 settings.

**Figure 6:**
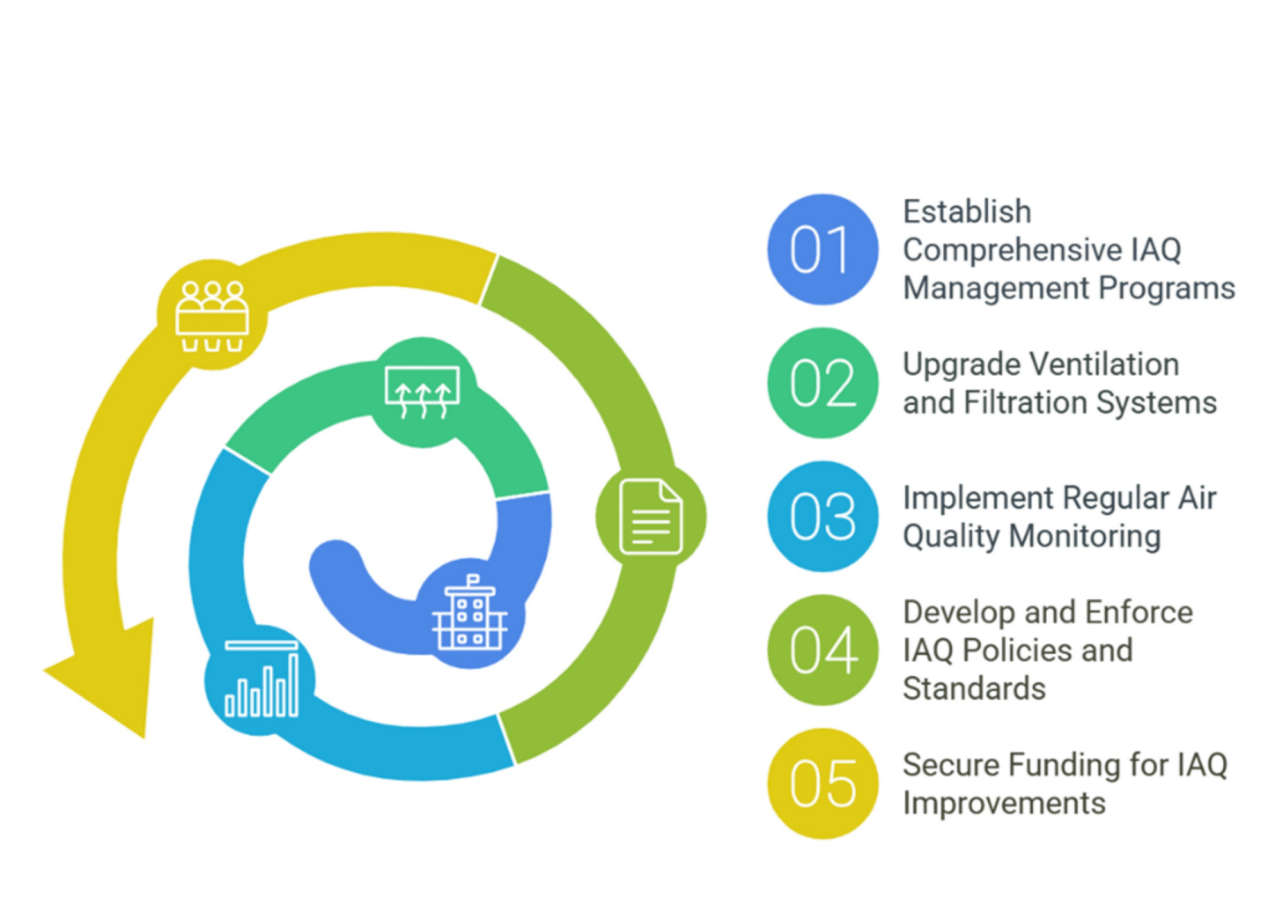
Improving indoor air quality in schools Image credit: Victor Ezeamii

Enhancing IAQ in schools requires a structured approach involving policy, technology, and infrastructure improvements. Figure 6 outlines key steps, including establishing IAQ management programs, upgrading ventilation systems, implementing regular monitoring, enforcing standards, and securing funding for long-term sustainability.

By implementing these policy recommendations, schools can create healthier indoor environments that support student well-being and academic success.

IAQ in schools is a critical determinant of student health, cognitive performance, and overall academic success. Our findings, discussed earlier, underscore the significant impact of air pollutants, such as CO₂, PM, VOCs, and allergens, on students' well-being. Poor IAQ has been linked to increased respiratory illnesses, including childhood asthma, and has been shown to negatively affect attention, memory, and decision-making abilities. Consequently, schools must prioritize ventilation improvements, real-time air quality monitoring, and the implementation of robust IAQ policies to foster a healthier learning environment.

Future research directions

This study reviewed and summarized available evidence on the adverse impact of poor indoor air quality on school children. It provides a clear background for future research. First, this study performed a quantitative analysis of pollutant concentration, ventilation rates, and other health metrics, making it difficult to measure the magnitude of improved ventilation. Second, confounding factors, such as nutrition and other health behaviors, were not considered. Despite significant progress in understanding the effects of IAQ on student health and cognitive function, several areas require further research. While current studies show a correlation between IAQ and student outcomes, long-term longitudinal studies are needed to determine causal relationships and the lasting effects of improved IAQ on academic achievement. Future research could focus on developing cost-effective and highly accurate IAQ sensors that can be widely implemented in schools while incorporating the impact of climate change. Rising temperatures, increased outdoor air pollution, and extreme weather events may further affect IAQ.

## Conclusions

The review highlights the following major insights: upgraded HVAC systems, HEPA filters, and hybrid ventilation systems have substantially reduced indoor air pollutants, lowered respiratory illness rates, and improved cognitive function among students. Adopting real-time IAQ monitoring technologies, including IoT-enabled sensors, allows for continuous assessment and timely intervention, preventing prolonged exposure to harmful pollutants. Poor air quality exacerbates asthma symptoms, leading to increased absenteeism and reduced academic performance. Effective IAQ management can help mitigate these effects and support student well-being. Also, elevated CO₂ levels and exposure to PM₂.₅ negatively impact student concentration, problem-solving, and standardized test performance. Schools with optimized air circulation and low pollutant levels report improved academic outcomes.

To ensure healthier IAQ in schools, institutions and policymakers should implement the following: adopting nationwide indoor air standards, to mandate proper ventilation in classrooms; investing in school infrastructure to modernize outdated ventilation systems and ensure compliance with IAQ standards; introducing training programs for school administrators, teachers, and students to raise awareness of air quality issues and encourage proactive measures; ensuring routine monitoring and reporting to ensure transparency and accountability in maintaining optimal air quality in schools.
